# Analysis of 51 proposed hypertrophic cardiomyopathy genes from genome sequencing data in sarcomere negative cases has negligible diagnostic yield

**DOI:** 10.1038/s41436-018-0375-z

**Published:** 2018-12-11

**Authors:** Kate L. Thomson, Elizabeth Ormondroyd, Andrew R. Harper, Tim Dent, Karen McGuire, John Baksi, Edward Blair, Paul Brennan, Rachel Buchan, Teofila Bueser, Carolyn Campbell, Gerald Carr-White, Stuart Cook, Matthew Daniels, Sri V. V. Deevi, Judith Goodship, Jesse B. G Hayesmoore, Alex Henderson, Teresa Lamb, Sanjay Prasad, Paula Rayner-Matthews, Leema Robert, Linda Sneddon, Hannah Stark, Roddy Walsh, James S. Ware, Martin Farrall, Hugh C. Watkins, NIHR BioResource – Rare Diseases Consortium

**Affiliations:** 1 Division of Cardiovascular Medicine, Radcliffe Department of Medicine, University of Oxford, Oxford, UK; 2 Oxford Medical Genetics Laboratories, Oxford University Hospitals NHS Foundation Trust, The Churchill Hospital, Oxford, UK; 3 National Heart and Lung Institute, Imperial College London, London, UK; 4 Oxford Centre for Genomic Medicine, Oxford University Hospitals NHS Foundation Trust, Oxford, UK; 5 Northern Genetics Service, Newcastle upon Tyne Hospitals NHS Foundation Trust, Newcastle, UK; 6 Cardiovascular Research Centre, Royal Brompton & Harefield Hospitals NHS Foundation Trust, London, UK; 7 King’s College London, Guy’s & St Thomas’ Hospital NHS Foundation Trust, King’s College Hospital NHS Foundation Trust, London, UK; 8 Guy’s & St Thomas’ Hospital NHS Foundation Trust, London, UK; 9 National Heart Research Institute Singapore, National Heart Centre Singapore, Singapore, Singapore; 10 Division of Cardiovascular & Metabolic Disorders, Duke-National University of, Singapore, Singapore; 11 MRC London Institute of Medical Sciences, Imperial College London, London, UK; 12 Department of Haematology, University of Cambridge, Cambridge Biomedical Campus, Cambridge, UK; 13 NIHR BioResource, Cambridge University Hospitals NHS Foundation, Cambridge Biomedical Campus, Cambridge, UK; 14 The Wellcome Trust Centre for Human Genetics, University of Oxford, Oxford, UK

**Keywords:** HCM, genetic testing, variant interpretation, VUS, evidence-based

## Abstract

**Purpose:**

Increasing numbers of genes are being implicated in Mendelian disorders and incorporated into clinical test panels. However, lack of evidence supporting the gene-disease relationship can hinder interpretation. We explored the utility of testing 51 additional genes for hypertrophic cardiomyopathy (HCM), one of the most commonly tested Mendelian disorders.

**Methods:**

Using genome sequencing data from 240 sarcomere gene negative HCM cases and 6229 controls, we undertook case-control and individual variant analyses to assess 51 genes that have been proposed for HCM testing.

**Results:**

We found no evidence to suggest that rare variants in these genes are prevalent causes of HCM. One variant, in a single case, was categorized as likely to be pathogenic. Over 99% of variants were classified as a variant of uncertain significance (VUS) and 54% of cases had one or more VUS.

**Conclusion:**

For almost all genes, the gene-disease relationship could not be validated and lack of evidence precluded variant interpretation. Thus, the incremental diagnostic yield of extending testing was negligible, and would, we propose, be outweighed by problems that arise with a high rate of uninterpretable findings. These findings highlight the need for rigorous, evidence-based selection of genes for clinical test panels.

## INTRODUCTION

Hypertrophic cardiomyopathy (HCM) is the most common inherited cardiac disorder, with a prevalence of approximately 1 in 500 individuals.^1^ It is an important cause of sudden death in athletes and young adults under 35 years of age. Familial HCM is predominantly caused by pathogenic variants in the genes encoding protein components of the cardiac sarcomere.^2^ Genetic testing for key HCM genes has been available for over a decade and is an integral part of patient care.^3,4^ Identification of a genetic cause of HCM in an individual can enable definitive identification of relatives at risk of HCM, who then need clinical assessment and follow up, and identification of relatives who can be discharged.

The genetics of HCM is complicated by genetic heterogeneity, incomplete penetrance, variable expressivity, and phenocopies, and current testing achieves a firm/confident genetic diagnosis in only around 40% of patients.^5,6^ An increasing number of genes are being asserted to cause this condition and incorporated into clinical genetic test panels.^7,8^ However, for many of these newly reported genes, lack of robust evidence supporting a causal role in HCM creates interpretation uncertainty. As such, an increasing number of variants are classified as variants of uncertain significance (VUS) that are not clinically actionable.^5,8^ This inevitably reduces the clinical utility and cost effectiveness of genetic testing. Of greater concern is the risk of incorrect interpretation, which could have serious consequences for at-risk family members who may be misdiagnosed (false positive result) or given false reassurance (false negative result). To minimize uncertainty and the potential for misdiagnoses, there is an urgent need to define explicitly the causal genes in this disorder and rectify previous erroneous findings.

Recent analyses using data from large clinical cardiomyopathy case series and reference control cohorts have enabled robust evaluation of the genes currently included on clinical gene panels, and highlighted the genes and classes of variants that can be reliably interpreted in a clinical setting.^6,9^

Using genome sequencing (GS) data from 240 probands in whom no pathogenic variants were detected in confirmed HCM genes, we extend these analyses to an additional 51 genes proposed for HCM testing in the Genomics England 100,000 Genomes Project. The frequency of rare variants in these genes was compared with 6229 reference controls from the National Institute for Health Research (NIHR) Bioresource for Rare Disease. Additionally, we perform case level analysis to review the evidence of pathogenicity for rare variants found in HCM cases, and classify each variant according to clinical guidelines.^10^

Our study was designed to contribute much needed insights into these gene–disease relationships to inform ongoing gene curation efforts.^11,12^

## MATERIALS AND METHODS

### Hypertrophic cardiomyopathy cases

Two hundred forty unrelated HCM patients were recruited to the NIHR Bioresource Rare Disease HCM project (hereafter BRRD) from May 2014 to September 2016. Eligibility criteria were:

- Age 18–70 years, or >70 years with a family history of HCM
- Clinical diagnosis of HCM made in a specialist inherited heart disease clinic within one of the following: Oxford University Hospitals National Health Service (NHS) Foundation Trust, Royal Brompton & Harefield NHS Foundation Trust, Guy's and St Thomas' NHS Foundation Trust or the Newcastle upon Tyne Hospitals NHS Foundation Trust
- Absence of “highly likely” or “likely” pathogenic variants within confirmed HCM genes following clinical genetic testing

Informed consent for genomic sequencing, and analysis of demographic, clinical, and family history data was obtained through the BRRD study (Research Ethics Committee reference 13/EE/0325) or earlier studies of the genetic basis of HCM. Where possible, documentation of HCM in a relative was confirmed through clinical or postmortem records. Prior to recruitment, genetic testing of a minimum of 13 HCM genes was undertaken by the Oxford Medical Genetics Laboratory (OMGL), a United Kingdom Accreditation Service (UKAS)-accredited clinical diagnostic laboratory, or the Royal Brompton & Harefield NHS Foundation Trust. This included testing of the eight well-established sarcomeric HCM genes (*MYBPC3*, *MYH7*, *TNNI3*, *TNNT2*, *MYL2*, *MYL3*, *ACTC1*, *TPM1*), genes for common differential diagnoses (*PRKAG2*, *GLA*, *FHL1*), and other more rarely associated, but validated, HCM genes (*CSRP3*, *PLN*).^5,6,9^

### Reference controls

Rare variant data from 6229 unrelated individuals, recruited to other rare disease projects within the BRRD, were used as controls in this analysis. Although formal clinical exclusion of HCM was not performed within our control cohort, the prevalence of HCM within this cohort is not expected to exceed that of the general population.

### Genome sequencing

Genome sequencing of cases and reference controls was undertaken by Illumina on behalf of the BRRD project. Principal component analysis was used to infer ethnicity (Online Methods 1.1 and 1.2).

### Gene selection

Candidate genes were derived from the Genomics England PanelApp (https://www.genomicsengland.co.uk and Online Methods 1.3). At the time of review (December 2016), 67 genes were listed for HCM, of which 16 were in OMGL’s HCM gene panel (*ACTC1*, *ACTN2*, *ANKRD1*, *CSRP3*, *FHL1*, *GLA*, *LAMP2*, *MYBPC3*, *MYH7*, *MYL2*, *MYL3*, *PLN*, *PRKAG2*, *TNNI3*, *TNNT2*, *TPM1*). We sought to examine the gene–disease relationships for the remaining 51 genes (Table S15). Illustrative power calculations for these analyses are shown in Table S1.

### Analysis protocol

Chromosome coordinates for each gene and transcript were obtained from Ensembl Genome build GRCh37 and combined into a single BED file (Table S2).

A range of open source bioinformatics tools were combined into a Python script to extract variants within regions of interest from the BRRD project merged VCF file. Tabix was used to extract variants from chromosome regions specified in the BED file. Variants within regions of interest were annotated using SNPEff. For each gene, SNPSift was used to retain variants for subsequent analysis that fulfilled the following criteria: PASS filter, a Genome Aggregation Database (gnomAD)^13^ global minor allele frequency (MAF) of ≤0.0001, and a SNPEff HIGH or MODERATE annotation . SNPSift was used to generate rare variant counts for cases and controls.

BAM files from all HCM cases were analyzed using SAMtools (https://www.htslib.org/) to produce depth of sequence coverage statistics for each coding nucleotide across all 51 candidate genes.

Fisher’s exact test and odds ratios (OR) were calculated for each gene by comparing the burden of rare variants (defined for the purposes of these analyses as variants with a MAF ≤0.0001 in gnomAD.^13^ Analyses were undertaken combining rare variants across all genes and for each gene individually. Prespecified separate analyses were undertaken focusing on missense, truncating (frameshift, nonsense, splice donor/acceptors), and nontruncating (missense, in-frame insertions and deletions) variants alone. As a control for technical factors, the prevalence of synonymous variants in each gene, the majority of which are not expected to be disease causing, was also assessed. Analyses were undertaken using R version 3.3.3 and R Studio Version 1.0.136.

### Variant classification

Classification of individual rare variants detected in HCM cases was undertaken using clinical guidelines.^10^ This incorporated disease specific knowledge relating to estimates of disease prevalence,^1^ penetrance, and our understanding of the genetic basis of HCM from previous analysis in large case cohorts.^5,6,14,15^

### Clinical sequencing validation

To confirm previous clinical genetic test findings, and to exclude the possibility of false negative findings, rare variant analysis was undertaken on 16 genes included in OMGL’s HCM gene panel (*ACTC1*, *ACTN2*, *ANKRD1*, *CSRP3*, *FHL1*, *GLA*, *LAMP2*, *MYBPC3*, *MYH7*, *MYL2*, *MYL3*, *PLN*, *PRKAG2*, *TNNI3*, *TNNT2*, *TPM1*), using the same methods and analytical pipeline as described above.

## RESULTS

### Clinical, demographic, and family history

Two hundred forty unrelated, sarcomere gene negative, HCM probands were included in these analyses. The mean age at specialist clinic evaluation was 53 years (SD 11.66, range 20–75). Thirty-two percent (77/240) of probands self-reported a family history of cardiomyopathy; for the 63 on whom we had data, 61 had at least one relative who was a patient of an inherited cardiac condition (ICC) service, or had a diagnosis of cardiomyopathy confirmed through postmortem records. For 14 probands who reported a positive family history, confirmatory data were not available. There was no difference in the mean age at genetic testing when comparing cases with and without a family history (*p*=0.14). Eighty-one percent of our HCM probands were male, compared with 40% of BRRD reference controls. The mean maximum left ventricular (LV) wall thickness was 1.8 cm (SD 0.43). Approximately 40% of cases were reported to have hypertension. The ethnicity of our case and control cohorts was broadly similar, with the majority (89% and 79% respectively) categorized as Northern European (non-Finnish) (Table S3). This reduces potential confounding effects due to differences in rare variant frequency between populations of different ethnicity.^16^

### Genes tested

**Fig. 1:**
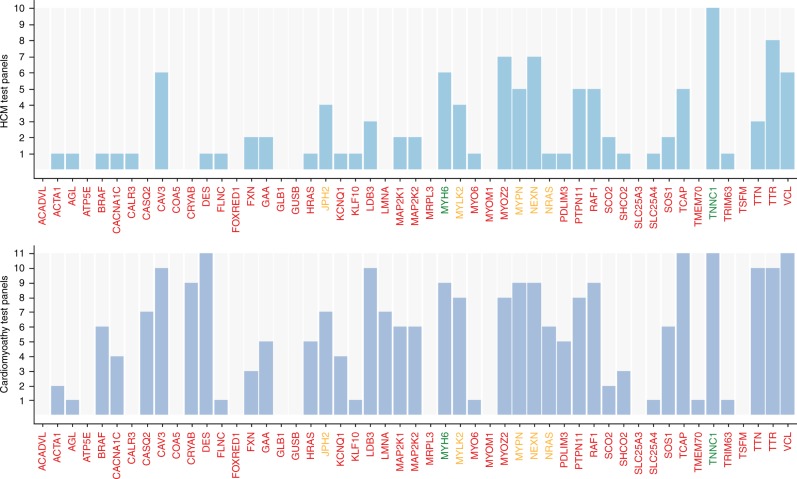
**Frequency of the 51 selected candidate genes in current commercial test panels.** All genes listed in the Genomics England hypertrophic cardiomyopathy (HCM) panel that were not in the Oxford Medical Genetics Laboratory (OMGL) clinical HCM test panel at the time of this study were selected for analysis. The bar chart displays the number of times each of the 51 selected candidate genes was included in a commercial test panel. Data was extracted from the National Center for Biotechnology Information (NCBI) Gene Tests website (October 2016) and Genetic Test Registry (December 2017). This included 10 clinical HCM panels and 11 clinical cardiomyopathy panels. Information on laboratories and available test panels is in Table S5. The gene labels are colored according to the review status as annotated by Genomics England, which gives an indication of the level of evidence supporting each gene–disease relationship: Green = high evidence, the gene is very likely be the cause of the disease and can be reported back to patients. Amber = moderate evidence, and should not yet be used for genome interpretation. Red = low evidence for a role in disease, or not suitable for clinical diagnosis at this time.

We examined the 51 genes listed for HCM on the Genomics England PanelApp (https://www.genomicsengland.co.uk) that had not been included in the prior clinical testing of these samples (Table S15). This list had been compiled from a range of established sources and from disease area experts. Each gene had been reviewed by experts throughout the scientific community and rated according to the level of evidence (Table S4). Of these, 2/51 (4%) were rated “Green” (definitive diagnostic grade genes), 5/51 (10%) “Amber” (moderate evidence), and 44/51 (86%) “Red” (low level of evidence). Approximately two-thirds of these genes are currently included in mainstream clinical test panels (Fig. 1 and Table S5).

### Gene coverage

The mean depth of sequence coverage across candidate gene coding regions was assessed to determine if there was sufficient sequencing data coverage to enable accurate variant calling. With the exception of one gene (myosin heavy chain 6 [*MYH6*]), the mean depth of sequence reads across coding nucleotides was at least 20× (Table S6 and Figure S1). Further investigations (data not shown) indicated that the reduced coverage observed in regions of *MYH6* was likely due to reads being discarded due to poor mapping quality of sequencing reads in regions that share high sequence homology with the myosin heavy chain 7 gene (*MYH7*).

### Gene level analysis

**Fig. 2:**
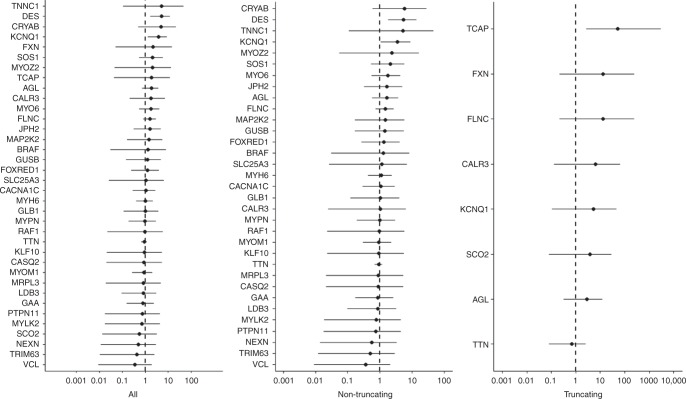
**Odds ratios with 95% confidence interval (CI) in hypertrophic cardiomyopathy (HCM) cases (*n*=240) compared with National Institute for Health Research Bioresource Rare Disease HCM project (BRRD) controls (*n*=6229).** All = all rare variants. Nontruncating = missense, in-frame insertions and deletions, and other variants annotated as MODERATE impact by SNPEff. . Truncating = variants predicted to result in a truncated transcript (nonsense, frameshift, canonical splice site). Rare is defined as gnomAD minor allele frequency (MAF) <0.0001. Data plotted using log10 scale. Genes in which no rare variants were detected in cases have not been plotted. A single truncating variant in *TCAP* was found in cases and no truncating variants were detected in controls; this result is not significant when corrected for multiple testing.

To examine the relationship between rare variants in the 51 candidate genes and HCM status, we compared the proportion of cases and controls with one or more rare variant across all genes, and for each gene individually, using Fisher’s exact test and odds ratio analyses. We expect differences in rare variant frequency arising due to variation in sequencing protocols and variant annotation to be minimal, as cases and controls were sequenced as part of the same study. Analysis was performed after combining all rare variants with a HIGH or MODERATE effect predicted by SNPEff (Table S7), and then separately for missense (Table S8), truncating (Table S9), and nontruncating (Table S10) variants; the results are summarized in Table 1 and Fig. 2.

**Table 1:** Results from case vs control analyses

	All variants				Non-truncating variants				Truncating variants			
Gene	Case	Control	FET p-value	OR^a^	Case	Control	FET p-value	OR^a^	Case	Control	FET p-value	OR^a^
*ACADVL* ^a^	0	0.010	0.734	0.41 (0.01–2.37)	0	0.009	0.725	0.45 (0.01–2.63)	0	0.001	0.263	3.69 (0.08–28.87)
*ACTA1* ^a^	0	0.001	0.291	3.23 (0.07–24.23)	0	0.001	0.263	3.69 (0.08–28.87)	0	0.0002	0.108	12.90 (0.22–248.29)
*AGL*	0.033	0.019	0.139	1.83 (0.76–3.80)	0.025	0.016	0.282	1.62 (0.57–3.71)	0.0083	0.003	0.169	2.90 (0.32–12.22)
*ATP5E* ^a^	0	0.000	0.108	12.90 (0.22–248.29)	0	0.000	0.108	12.90 (0.22–248.29)	0	0	–	–
*BRAF*	0.004	0.003	0.549	1.30 (0.03–8.19)	0.004	0.003	0.549	1.30 (0.03–8.19)	0	0	–	–
*CACNA1C*	0.017	0.016	0.791	1.06 (0.28–2.84)	0.017	0.015	0.788	1.08 (0.29–2.90)	0	0.0003	0.141	8.61 (0.16–107.59)
*CALR3*	0.008	0.005	0.320	1.80 (0.21–7.17)	0.004	0.004	1	1.04 (0.03–6.40)	0.0042	0.0006	0.172	6.51 (0.13–66.21)
*CASQ2*	0.004	0.005	1	0.86 (0.02–5.25)	0.004	0.005	1	0.89 (0.02–5.44)	0	0.0002	0.108	12.90 (0.22–248.29)
*CAV3* ^a^	0	0.001	0.263	3.69 (0.08–28.87)	0	0.001	0.263	3.69 (0.08–28.87)	0	0	–	–
*COA5* ^a^	0	0.001	0.174	6.46 (0.13–65.67)	0	0.000	0.141	8.61 (0.16–107.59)	0	0.0002	0.108	12.90 (0.22–248.29)
*CRYAB*	0.008	0.002	0.082	4.75 (0.51–21.93)	0.008	0.001	0.061	5.80 (0.61–28.26)	0	0.0003	0.141	8.61 (0.16–107.59)
*DES*	0.025	0.005	0.003	4.96 (1.68–12.19)	0.025	0.005	0.002	5.48 (1.84–13.60)	0	0.0005	0.174	6.46 (0.13–65.67)
*FLNC*	0.054	0.034	0.106	1.61 (0.83–2.87)	0.050	0.034	0.203	1.49 (0.75–2.72)	0.0042	0.0003	0.107	13.01 (0.22–250.28)
*FOXRED1*	0.013	0.010	0.735	1.26 (0.25–3.90)	0.013	0.009	0.494	1.35 (0.27–4.18)	0	0.0006	0.204	5.16 (0.11–46.40)
*FXN*	0.004	0.002	0.389	2.17 (0.05–14.76)	0	0.002	0.367	2.35 (0.05–16.26)	0.0042	0.0003	0.107	13.01 (0.22–250.28)
*GAA*	0.013	0.016	1	0.78 (0.16–2.39)	0.013	0.014	1	0.87 (0.18–2.67)	0	0.002	0.367	2.35 (0.05–16.26)
*GLB1*	0.008	0.008	1	1.00 (0.12–3.8)	0.008	0.008	0.719	1.04(0.12–3.99)	0	0.0003	0.141	8.61 (0.16–107.59)
*GUSB*	0.008	0.007	0.674	1.27 (0.15–4.93)	0.008	0.006	0.651	1.45 (0.17–5.67)	0	0.0008	0.234	4.30 (0.09–35.67)
*HRAS* ^a^	0	0.002	0.457	1.72 (0.04–11.26)	0	0.002	0.391	2.15 (0.05–14.64)	0	0.0005	0.174	6.46 (0.13–65.67)
*JPH2*	0.013	0.008	0.448	1.56 (0.31–4.90)	0.013	0.008	0.438	1.63 (0.32–5.12)	0	0.0003	0.141	8.61 (0.16–107.59)
*KCNQ1*	0.025	0.007	0.009	3.69 (1.27–8.82)	0.021	0.006	0.021	3.47 (1.06–8.93)	0.0042	0.0008	0.203	5.21 (0.11–46.78)
*KLF10*	0.004	0.005	1	0.89 (0.02–5.44)	0.004	0.005	1	0.93 (0.02–5.65)	0	0.0002	0.108	12.90 (0.22–248.29)
*LDB3*	0.008	0.010	1	0.81 (0.01–3.08)	0.008	0.010	1	0.85 (0.10–3.24)	0	0.0005	0.174	6.46 (0.13–65.67)
*LMNA* ^a^	0	0.007	1	0.58 (0.01–3.46)	0	0.007	1	0.58 (0.01–3.46)	0	0	–	–
*MAP2K1* ^a^	0	0.002	0.457	1.72 (0.04–11.26)	0	0.002	0.457	1.72 (0.04–11.26)	0	0	–	–
*MAP2K2*	0.008	0.006	0.651	1.45 (0.17–5.67)	0.008	0.006	0.402	1.49(0.17–5.85)	0	0.0002	0.108	12.90 (0.22–248.29)
*MRPL3*	0.004	0.005	1	0.81(0.02–4.89)	0.004	0.005	1	0.89 (0.02–5.44)	0	0.0005	0.174	6.46 (0.13–65.67)
*MYH6*	0.029	0.029	0.845	1.02 (0.40–2.17)	0.029	0.027	0.837	1.09 (0.43–2.33)	0	0.002	0.414	1.98 (0.05–13.31)
*MYLK2*	0.004	0.006	1	0.72 (0.02–4.31)	0.004	0.006	1	0.76 (0.02–4.59)	0	0.0003	0.141	8.61 (0.16–107.59)
*MYO6*	0.021	0.012	0.227	1.72 (0.54–4.26)	0.021	0.012	0.218	1.77 (0.55–4.38)	0	0.0003	0.141	8.61 (0.16–107.59)
*MYOM1*	0.021	0.024	1	0.86 (0.27–2.09)	0.021	0.022	1	0.94 (0.30–2.28)	0	0.002	0.414	1.98 (0.05–13.31)
*MYOZ2*	0.004	0.002	0.411	2.00 (0.05–13.42)	0.004	0.002	0.365	2.36 (0.05–16.39)	0	0.0003	0.141	8.61 (0.16–107.59)
*MYPN*	0.013	0.013	1	0.97 (0.20–2.98)	0.013	0.013	1	0.99 (0.20–3.02)	0	0.0002	0.108	12.90 (0.22–248.29)
*NEXN*	0.004	0.008	0.723	0.50 (0.01–2.92)	0.004	0.007	1	0.56 (0.01–3.32)	0	0.001	0.263	3.69 (0.08–28.87)
*NRAS* ^a^	0	0.001	0.317	2.87 (0.07–20.84)	0	0.001	0.317	2.87 (0.07–20.84)	0	0	–	–
*PDLIM3* ^a^	0	0.004	0.615	1.07 (0.03–6.64)	0	0.003	0.568	1.23 (0.03–7.69)	0	0.0005	0.174	6.46 (0.13–65.67)
*PTPN11*	0.004	0.006	1	0.74 (0.02–4.45)	0.004	0.006	1	0.74 (0.02–4.45)	0	0	–	–
*RAF1*	0.004	0.004	1	0.96 (0.02–5.88)	0.004	0.004	1	0.96 (0.02–5.88)	0	0	–	–
*SCO2* ^a^	0.004	0.008	1	0.55 (0.01–3.25)	0	0.006	1	0.63 (0.02–3.73)	0.0042	0.001	0.261	3.72 (0.08–29.12)
*SHOC2* ^a^	0	0.004	1	0.99 (0.02–6.08)	0	0.004	1	0.99 (0.02–6.08)	0	0	–	–
*SLC25A3*	0.004	0.004	0.612	1.08 (0.03–6.69)	0.004	0.004	0.597	1.13 (0.03–7.01)	0	0.0002	0.108	12.90 (0.22–248.29)
*SLC25A4* ^a^	0	0.001	0.343	2.58 (0.06–18.27)	0	0.001	0.343	2.58 (0.06–18.27)	0	0	–	–
*SOS1*	0.017	0.008	0.139	2.09 (0.54–5.78)	0.017	0.008	0.139	2.09 (0.54–5.78)	0	0	–	–
*TCAP* ^a^	0.004	0.002	0.433	1.86 (0.04–12.30)	0	0.002	0.457	1.72 (0.04–11.26)	0.0042	0	0.004	51.81 (2.69–2989.44)
*TMEM70* ^a^	0	0.003	0.584	1.17 (0.03–7.31)	0	0.003	0.497	1.52 (0.04–9.75)	0	0.0008	0.234	4.30 (0.09–35.67)
*TNNC1*	0.004	0.001	0.203	5.2 (0.11–46.78)	0.004	0.001	0.203	5.21 (0.11–46.78)	0	0	–	–
*TRIM63*	0.004	0.010	0.727	0.44 (0.01–2.56)	0.004	0.008	0.723	0.5 (0.01–2.92)	0	0.001	0.291	3.23 (0.07–24.23)
*TSFM* ^a^	0	0.005	1	0.76 (0.02–4.55)	0	0.004	1	0.92 (0.02–5.61)	0	0.001	0.263	3.69 (0.08–28.87)
*TTN*	0.388	0.413	0.463	0.90 (0.68–1.18)	0.379	0.401	0.546	0.91 (0.69–1.20)	0.0083	0.012	1	0.69 (0.08–2.61)
*TTR* ^a^	0	0.001	0.263	3.69 (0.08–28.87)	0	0.001	0.263	3.69 (0.08–28.87)	0	0	–	–
*VCL*	0.004	0.012	0.532	0.34 (0.01–1.99)	0.004	0.011	0.524	0.36 (0.01–2.11)	0	0.0006	0.204	5.16 (0.11–46.40)

Case frequency (BRRD HCM n=240) and Control frequency (BRRD non-HCM n=6229), Fishers Exact Test (FET) p value and Odds Ratios with 95% Confidence intervals, for all rare variants combined and for Non-truncating variants and Truncating variants. Rare is defined as gnomAD MAF <0.0001. Non-truncating includes missense, in-frame insertions/deletions and other variants annotated as “MODERATE” impact by SNPEff. Bonferroni corrected p=0.0009 (0.05/51). ^a^For genes where the case allele count was zero, 0.5 was added to all cells before calculating the odds ratio (Halden contingency correction)

Overall, combining data from all genes, there was no significant difference in the proportion of HCM cases and BRRD controls carrying one or more rare variant (all genes and variants: OR 1.12 [95% CI 0.86–1.45], *p*=0.43, nontruncating variants: OR 1.11 [95% CI 0.86–1.43], *p*=0.47, and truncating variants: OR 1.19 [95% CI 0.62–2.27], *p*=0.59, Table S11).

In the analyses of individual genes, and allowing for multiple testing (Bonferroni corrected significance threshold *p* <0.05/51, i.e., <0.001), a significant case excess of rare variation was not detected in any gene. Additionally, there was no significant difference in the proportion of rare synonymous variants in HCM cases compared with BRRD controls, indicating comparable sensitivity for rare variant detection (Table S16).

### Variant level analysis

To determine whether there was evidence to support the pathogenicity of any individual variant, each variant was reviewed and classified according to clinical guidelines.^10^ A total of 186 rare variants were detected in 36 of the 51 candidate genes in cases. In 15 genes, no rare variants were found in cases; rare variants were detected in controls in all 51 genes (Table S12). A single variant was classified as likely pathogenic (LP) (0.5%), and 9 (4.8%) were classified as benign or likely benign (B/LB). Most of the variants (176/186, 94.6%) were classified as VUS (Tables S13 and S14). Almost half of the VUS were in the titin (*TTN*) gene (87/176, 49.4%).

The one variant classified as likely pathogenic disrupted a splice site in the *FLNC* gene, c.6004+2T>C. This variant was absent from the BRRD control samples and the Genome Aggregation Database (gnomAD, http://gnomad.broadinstitute.org) (American College of Medical Genetics and Genomics [ACMG] criteria PM2 [refs.^10,14^]). Bioinformatic analysis predicts that this nucleotide substitution would disrupt the splice donor site, causing a frameshift of the amino acid sequence and premature termination of translation (Figure S2). Variants predicted to lead to premature termination of translation in *FLNC* have been detected in individuals with dilated, arrhythmogenic, and restrictive cardiomyopathies,^17–20^ although this class of variant has not been widely reported in HCM patient cohorts^17,21,22^ (ACMG criteria PVS1_Strong^10,14^).

### Case level analysis

To assess the clinical utility of extended candidate gene analyses in our patient cohort, we considered the outcomes of testing in each proband. We found high levels of uncertain findings, with 54% (129/240) of cases reported to have at least one VUS and more than one VUS in 15% (35/240) of cases. The majority of rare variants were detected in the *TTN* gene; excluding *TTN* from analyses, the proportion of probands with at least one VUS was 34% (81/240), with 5% (11/240) having more than one VUS (Figure S3).

Analysis of the genes currently included on the OMGL HCM gene panel confirmed all prior findings, and did not detect any additional variants. Since initial clinical sequencing, one variant (*MYH7* c.5135G>A (p.Arg1712Gln) had been reclassified, from VUS to likely pathogenic.

### *FLNC*

The likely pathogenic *FLNC* c.6004+2T>C variant was detected in an individual with a clinical diagnosis of HCM diagnosed at less than 25 years of age, who remains asymptomatic with a maximum LV wall measurement of 1.7 cm, with good LV systolic function at age 40. A raised creatine kinase level was observed at diagnosis; however, two subsequent measurements were normal. This variant was present in the proband’s deceased father, who was diagnosed with HCM/restrictive cardiomyopathy (RCM) at age 40, and developed progressive heart failure requiring cardiac transplantation at age 49. As no additional relatives were known to be affected, further segregation analysis was not possible.

## DISCUSSION

**Fig. 3:**
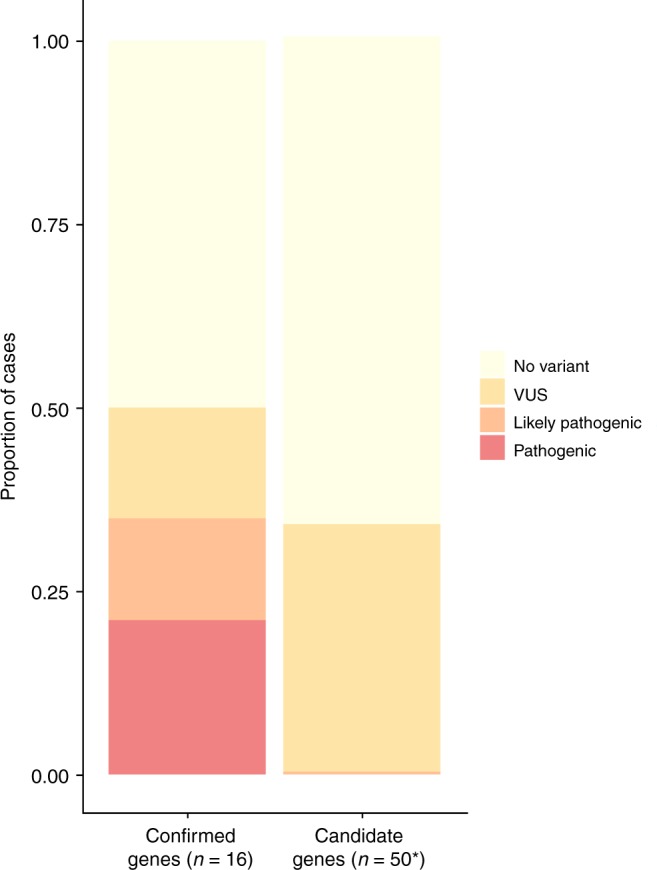
**Proportions of cases with different classes of reportable variants.** Confirmed genes: proportion of cases with a reportable variant in confirmed hypertrophic cardiomyopathy (HCM) genes (*n*=16). Comparison data from Oxford Medical Genetics Laboratory clinical HCM 16-gene panel from HCM cases (*n*=1082) referred for genetic testing from period January 2014 to September 2015. Candidate genes: proportion of cases with a reportable variant in the genes tested in this study in 240 HCM cases in whom no likely pathogenic or pathogenic variant was detected in confirmed genes. *N=50 variants detected in the *TTN* gene are not shown (*TTN* OR was 0.9 [95%CI 0.68–1.18]. *TTN* VUS are found in an additional 20% of cases). Note that for the 16 confirmed genes, case excess data indicate that the majority of VUS will in fact be disease causing, whereas the absence of any excess in cases in the 50 candidate genes indicates that the vast majority of VUS will not be disease causing. *CI* confidence interval, *OR* odds ratio, *VUS* variants of uncertain significance

We report the results of a rare variant analysis in 51 proposed candidate HCM genes in a sarcomere gene panel negative HCM cohort. A single variant detected in the *FLNC* gene was classified as likely pathogenic. We found no evidence to suggest that any of the other 186 rare variants detected in our case series were disease causing. Thus, there was hardly any additional yield of clinically actionable findings, whereas inclusion of these 51 genes resulted in a marked increase in the proportion of patients with uncertain, clinically unactionable results (Fig. 3).

4

Integral to these analyses was the availability of genome sequencing data from the HCM cohort, along with extensive control data sequenced as part of the same study. Cases were recruited from a cohort of patients with a clinical diagnosis of HCM in whom no pathogenic variants were found in confirmed HCM genes. This cohort should therefore be enriched for non-sarcomeric genetic forms of HCM making it an ideal subgroup in which to explore the contribution of putative candidate genes. To our knowledge this is the largest HCM GS cohort reported to date.

Overall, we found no evidence to suggest that rare variants in the 51 candidate genes analyzed are prevalent causes of HCM. This does not exclude the possibility that a small proportion of rare variants in these genes could be disease causing; however, it does suggest that the majority are not penetrant pathogenic variants. Indeed, in genes that are not themselves validated as HCM genes, it is likely that the vast majority of, if not all, individual VUS are not disease causing. This is a critical consideration for laboratories considering these genes for inclusion in clinical test panels. If only a small fraction of rare variants are expected to be pathogenic in a given gene, then results of testing that gene will only occasionally be interpretable. Only in large families with extensive segregation data, or cases with clear additional clinical features (i.e., specifying a given phenocopy), could there be an actionable outcome. This was evident in that all but one of the rare variants detected in our cases were classified as VUS.

In the absence of a clear clinical or genetic epidemiological indication for including these genes in HCM test panels, the potential for harm becomes paramount. For current clinical test panels, which focus on the core well-established HCM genes, approximately one-third of variants are classified as VUS and around 15% of patients are reported to have at least one VUS.^6,23^ However, in these validated genes case excess data indicate that the majority of variants classed VUS are in fact pathogenic,^6^ so over time the clinical yield from these core genes is set to grow and the proportion of VUS set to fall. The contrasting outcome of testing large numbers of unvalidated candidate disease genes was apparent in our cohort: over 99% of rare variants were VUS with a concomitant increase in the proportion of patients reported to have a VUS (Fig. 3). Importantly, the absence of any excess in cases in the 51 candidate genes indicates that the vast majority of these VUS will not be disease-causing. Including these genes in clinical test panels would have a significant adverse impact on patient management because although they are not considered clinically actionable, VUS are usually included in clinical reports, and often elicit further clinical and genetic family studies. These can be costly both in terms of clinical resources and patient anxiety, and often fail to resolve variant pathogenicity, a particular problem in late-onset, incomplete penetrance conditions such as HCM. Of greater concern is the potential for erroneous interpretation and the impact of false positive results for at-risk family members leading to misdiagnosis and inappropriate therapy, or false negative results, leading to inappropriate discharge from follow up.^24^

There is increased awareness of the issues surrounding variant interpretation particularly in the context of broad-based candidate gene analyses^25–27^ and a drive toward more consistent and evidence-based approaches to classification.^10,28^ Thus, the results of this study provide much needed empirical data that will inform current gene curation initiatives, such as those led by the National Institutes of Health (NIH) Clinical Genome Resource (ClinGen)^11,12^ and Genomics England. These efforts aim to confirm the genes definitively linked to inherited disorders and enable robust evidence-based selection of genes for clinical testing. Evidence-based approaches to genetic testing are critical in a clinical setting, and will become increasingly important as clinical exome and genome sequencing become more mainstream.^29,30^

Certainly, the findings in this study indicate that in individuals with a clinical diagnosis of HCM, testing beyond confirmed HCM genes is unlikely to increase the yield of clinically actionable results and therefore that exome- or genome-wide approaches will have limited utility in this setting. This is supported by a recent study that explored the utility of genome sequencing in HCM patients;^31^ here, the additional pathogenic variants were in the known HCM genes, or in definitive syndromic genes where isolated left ventricular hypertrophy (LVH) is a reported feature, thus the gains were not related to the broadening of testing to include genes with limited or no prior evidence of causality.

There are a number of possible reasons for the striking failure of validation of a broad range of genes implicated in HCM. We may not have had sufficient power to validate genes where only a small fraction of variants are pathogenic; however, analysis on this scale would indicate a significant excess of variants in most of the validated HCM genes. Moreover, if many of the genes tested do harbor occasional pathogenic variants, we would have expected to see some evidence of an overall burden across the panel, even if not in a specific gene. It remains possible that there are other, as yet undiscovered, Mendelian disease genes for HCM, but that current knowledge did not allow their identification through a candidate gene approach. However, a more likely interpretation is that the majority of sarcomere negative HCM probands do not have a monogenic disorder. This is supported by the observation that in these sarcomere gene negative cases we quite often did see evidence of familial disease but typically only in first degree relatives in small nuclear families (i.e., where multiple variants may be shared); this is in contrast to families with pathogenic variants in sarcomere genes, which generally behave like typical monogenic disorders, often with affected relatives across large extended pedigrees.^32,33^

Rare variants in candidate genes that are not supported by robust analyses, such as those in the current study, may be proposed as potentially significant modifier genes/variants. However, if that were the case then one would still expect to see an excess burden in affected cases, as is seen for modifier alleles in well-defined oligogenic diseases^34^ and complex traits.^35^ Finally, some of the genes on the panel are bona fide disease genes for other inherited cardiac conditions. Our findings in no way contradict this but, instead, indicate that testing these genes outside those clinically defined disorders is not useful.

In conclusion, consolidated analysis of sequence data from large case series is needed for comprehensive and robust assessment of candidate disease genes and putative causal variants; an advantage of GS data is that it enables assessment of both those genes already implicated in the literature and others that may follow. This will offset the recent trend of inclusion of putative disease genes despite insufficient evidence and ensure that genetic testing strategies are optimally effective and clinically beneficial.

## Electronic supplementary material

Supplementary Table
